# Exosomes in mammals with greater habitat variability contain more proteins and RNAs

**DOI:** 10.1098/rsos.170162

**Published:** 2017-04-26

**Authors:** Kazuhiro Takemoto, Miku Imoto

**Affiliations:** Department of Bioscience and Bioinformatics, Kyushu Institute of Technology, Iizuka, Fukuoka 820-8502, Japan

**Keywords:** habitat variability, genomics, exosome, environmental adaptation

## Abstract

Factors determining habitat variability are poorly understood despite possible explanations based on genome and physiology. This is because previous studies only focused on primary measures such as genome size and body size. In this study, we hypothesize that specific gene functions determine habitat variability in order to explore new factors beyond primary measures. We comprehensively evaluate the relationship between gene functions and the climate envelope while statistically controlling for potentially confounding effects by using data on the habitat range, genome, body size and metabolism of various mammals. Our analyses show that the number of proteins and RNAs contained in exosomes is predominantly associated with the climate envelope. This finding indicates the importance of exosomes to habitat range expansion of mammals and provides a new hypothesis for the relationship between the genome and habitat variability.

## 1. Introduction

The evolution of species habitat variability is an important topic for a wide range of research fields, particularly in the context of predictions related to biodiversity and climate change [1–3]. Thus, understanding factors that determine habitat use is relevant for advancing these fields. In particular, it is important to identify the molecular mechanisms that contribute to determining a species habitat range, because the behaviour of a species may result from complex biological systems.

Genetic studies are useful in this context. Specifically, previous studies have shown the importance of genomic properties. For example, genome size and the number of total genes increase with environmental variability because organisms need more functional (e.g. metabolic) genes in order to adapt to changing environments (e.g. nutrient variability) [4,5]. Genetic variation indicates a species ability to adapt to and exploit new environments [6]. Moreover, recent studies have reported that gene duplication (the number of duplicated genes) positively contributes to habitat variability in flies [7] and in mammals [8]. This has derived from the proposed importance of gene duplication to increasing biological robustness and evolvability [9], which are, themselves, related to habitat variability.

However, these previous studies have limitations. In particular, they focused only on primary measures of genomic information, such as genome size and the number of genes. The association between gene duplication and habitat variability was concluded in Euarchontoglires, but not in all mammals whose genomes were available [8]. In order to explore new factors beyond these primary measures, a more careful investigation is required. In this study, we hypothesized that specific functional categories determine habitat variability rather than primary genomic measures. As previous studies [10–14] have mentioned, the genome encodes several types of biological functions. The relationship between the number of functional genes and primary genomic measures (e.g. genome size and the number of total genes) differ according to functional category (e.g. as defined by Gene Ontology [15]). This suggests that genes in a specific functional category increase or decrease to acquire biological functions.

Within this context, we also need to consider the effect of metabolic rate and body size as it remains possible that the relationships between genomic measures and species habitat variability are spurious correlations, which result from the difference between metabolic rate and body size. Mass-specific metabolic rate, *B*_c_, is the oxygen consumption rate per unit body mass and is roughly equal to the rate at the cellular level. It correlates to the number of total genes, the number of genes in a specific functional category [12,16] and also to genetic variation [17]. This rate differs substantially among animal species and is specifically, negatively associated with body size, *M*. This is known as Kleiber's Law or the allometric scaling of metabolic rate [18–20]: *B*_c_ ∝ *M*^−1/4^. The mutation rate and generation time affect genetic events (e.g. gene duplication and genetic variation). The mutation rate decreases with body size [21,22] because of a decrease in mass-specific metabolic rate (or avoidance of oxidative DNA damage [23]) and an increase in the generation time [24]. These facts indicate the importance of taking into account (i.e. correcting for) the metabolic rate when evaluating relationships with genomic measures [16]. More importantly, metabolic rate and body size determine animal space use because they are related to the energy required for moving [25]. Space use indicates the potential of habitat range expansion. This fact suggests that metabolic rate and body size also influence species habitat variability.

We aimed to comprehensively explore the functional categories of genes beyond earlier (possible) factors for explaining habitat variability in mammals. We collected the genomic data, spatial data, metabolic rates and body sizes of mammals from databases and the literature. We then evaluated the relationships between gene functions and habitat variability by considering the effects of other factors, such as primary genomic measures and metabolic rate, by using phylogenetic comparative methods.

## 2. Material and methods

### 2.1. Habitat variability

Our method was similar to that reported in a previous study [8] which considered the climate envelope as a score of habitat variability. The climate envelope indicates the temperature range, precipitation and other climatic parameters in the habitat area of mammals and is calculated based on 19 bioclimatic variables (www.worldclim.org/bioclim) using principal component analysis (PCA). The spatial data on the habitat areas of terrestrial mammals were downloaded from the International Union for Conservation of Natural Resources Red List website (www.iucnredlist.org/technical-documents/spatial-data) on 26 August 2016. Climate data within habitat areas at a spatial resolution of 10 min of a degree were downloaded from the WorldClim database (v. 1.4, release 3) [26] (www.worldclim.org) using R software (v. 3.3.1) (www.R-project.org) and R-package *raster* (v. 2.5–8). The PCA showed that the first two principal components (PCs) explained 99.6% of the total variance; the contributions of PC1 and PC2 were 98.1% and 1.5%, respectively. We considered 14 715 (=981 × 15) cells to weigh the relative contributions of PC1 and PC2 when estimating the climate envelope. The climate envelope was defined as the number of overlapping points in the 14 715 cell grids.

We also considered a different definition of habitat variability: habitat diversity. According to a previous study [8], habitat diversity is defined by the Köppen–Geiger climate classification in species habitat areas using the Brillouin Index, a diversity measure which is robust to sample size. The Köppen–Geiger climatic classification map (data observed between 1976 and 2000) required to calculate the habitat diversity was downloaded online (koeppen-geiger.vu-wien.ac.at/shifts.htm) on 3 January 2016.

### 2.2. Genome, metabolic rate and body mass

We selected mammalian species whose genomes were available in the Kyoto Encyclopaedia of Genes and Genomes (KEGG) database [27] for use in the KEGG BRITE Functional Hierarchy (see §2.3). The genome statistics (i.e. genome size, *G* (bp) and the number of total genes, *N*_g_) and protein sequence data of the species were obtained from the KEGG database on 25 August 2016. The number of duplicated genes, *N*_d_, was estimated according to a previous study [8]. We performed an all-against-all Basic Local Alignment Search Tool search for all protein sequences. Homologous genes in the same species whose *E* < 10^–5^ and query coverage > 30% were defined as duplicated genes. We collected data on mass-specific metabolic rates *B*_c_ (W g^−1^) and body mass *M* (g) from our previous studies [12,16]. The data for 32 mammalian species are available in the electronic supplementary material, table S1.

### 2.3. Functional categories of genes

In our previous studies [12,16], we used the KEGG BRITE Functional Hierarchy [27] (www.kegg.jp/kegg/brite.html) for calculating the number of genes in functional categories (NOGFs). In this study, we did not consider Gene Ontology (GO) [15] as a definition of functional category because there were fewer organisms whose GO annotations were completed, compared to the KEGG BRITE Functional Hierarchy. We obtained data on the relationships between functional category and gene identifiers of species, S, from the KEGG FTP website (ftp.bioinformatics.jp/kegg/brite/organisms/S/) on 25 August 2016, where S corresponds to the KEGG organism identifier (www.genome.jp/kegg/catalog/org_list.html). In total, we considered 384 functional categories (electronic supplementary material, table S1).

### 2.4. Statistical analysis

To evaluate the contribution of each factor to the climate envelope, we performed a phylogenetic multivariate analyses using R (v. 3.3.2). Ideally, we would have used a direct phylogenetic regression model (e.g. phylogenetic generalized least squares) for all 389 explanatory variables. However, we could not perform such an analysis in this study because of the combinatorial explosion in model selection and the multicollinearity that mainly arises from gene overlap among functional categories or the hierarchical organization of functional categories [12,16]. In fact, we were unable to perform the phylogenetic generalized least squares using the *gls* function in the R-package *nlme* (v. 3.1–128) because of singularities in the regression model. As mentioned in our previous study [12], this is a particular problem with GO analyses. To avoid this problem as much as possible, we considered parameter selection using a phylogenetic version of the least absolute shrinkage and selection operator (LASSO) method and completed the statistical analysis using phylogenetic generalized least squares.

To identify candidates of functional categories associated with the climate envelope, we first considered the LASSO method that would be useful both for parameter selection and for regularization in order to increase the interpretability of the regression model for finding significant variables [28]. To remove any phylogenetic effects from the association between biological variables, phylogenetically independent contrasts (PICs) of the variables were computed from phylogenetic trees using the *pic* function in the R-package *ape* (v. 3.5). According to previous studies [8,16,29] (and also for comparison with the previous study [8]), the mammalian phylogenetic tree (electronic supplementary material, figure S1) used in the analyses was constructed using the matrix extracellular phosphoglycoprotein precursor gene, downloaded from the KEGG database on 29 August 2016. We performed the parameter selection using the LASSO method using the *cv.glmnet* and *glmnet* functions in the R-package *glmnet* (v. 2.0.5).

To control for confounding effects, we directly performed a phylogenetic regression analysis using the *gls* function of the R-package *nlme* and the phylogenetic tree. Specifically, we constructed full models encompassing number of genes in exosomes (NOGFs) of the identified candidates, *N*_g_, *N*_d_, *G*, *M* and *B*_c_, and selected the best model using the sample-size-corrected version of the Akaike information criterion (AICc). To avoid model selection bias, we also adopted a model-averaging approach [30,31]. We obtained averaged models in the top 95% confidence set using the *model.avg* function in the R-package *MuMIn* (v. 1.15.6). Climate envelope was square-root transformed for all analyses. The contribution (i.e. non-zero estimate) of NOGF to the climate envelope was completed only when the associated *p* < 0.01.

## 3. Results

**Figure 1.**
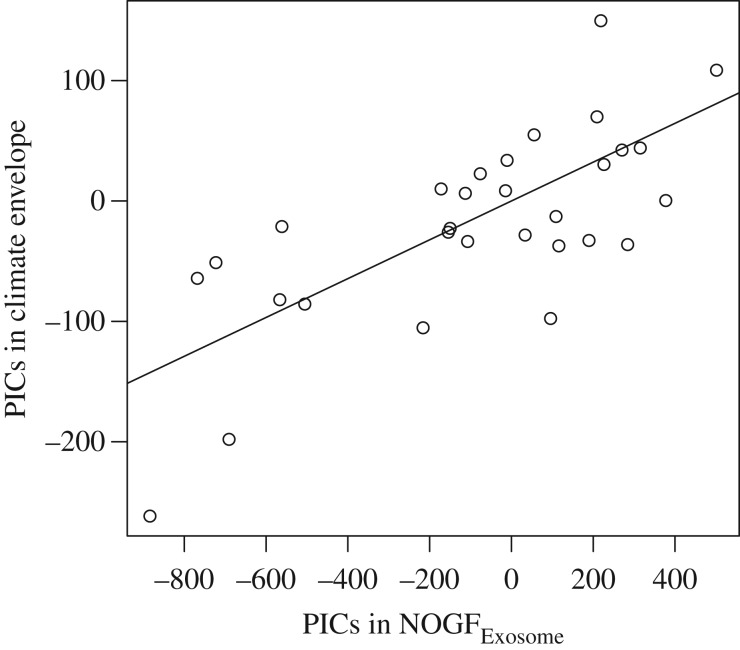
A scatter plot of PICs in the number of genes in the functional category *Exosome* (NOGF_Exosome_) versus PICs in the climate envelope. The solid line is the regression line (*R*^2^ = 0.54, *p* = 2.4 × 10^–6^).

**Figure 2.**
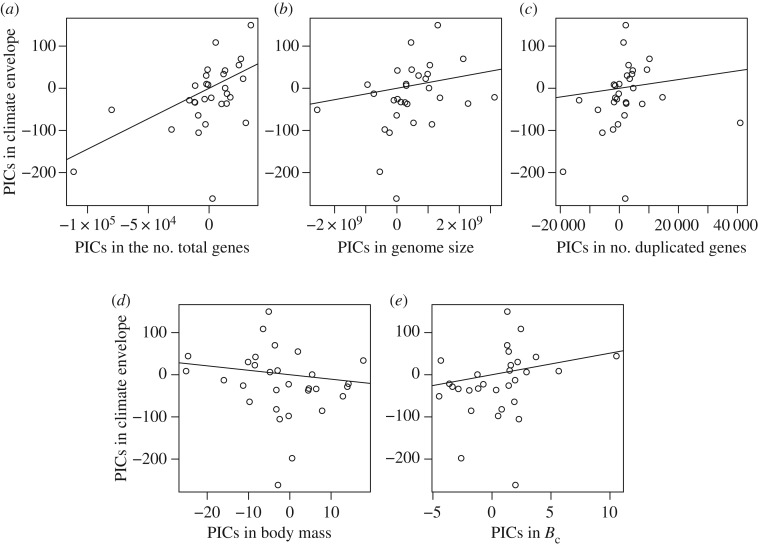
Scatter plots of PICs in genomic measures versus PICs in the climate envelope. (*a*) Genome size (*R*^2^ = 0.27, *p* = 0.0030), (*b*) number of total genes (*R*^2^ = 0.034, *p* = 0.32), (*c*) number of duplicated genes (*R*^2^ = 0.015, *p* = 0.51), (*d*) body mass (*R*^2^ = 0.017, *p* = 0.48), and (*e*) mass-specific metabolic rate (*R*^2^ = 0.038, *p* = 0.30).

After performing the LASSO method, only the functional category of *Exosome* was selected (electronic supplementary material, table S2). In particular, we found that the number of genes in exosomes (NOGF_Exosome_) was positively associated with the climate envelope (figure 1; the coefficient of determinant in the linear regression *R*^2^ = 0.54, the associated *p*-value = 2.4 × 10^–6^). As shown in figure 2, however, the climate envelope was still positively correlated with the number of total genes (figure 2*a*; *R*^2^ = 0.27, *p* = 0.0030); whereas the correlation between the climate envelope and the following other measures were hardly conclusive: genome size (figure 2*b*; *R*^2^ = 0.034, *p* = 0.32), number of duplicated genes (figure 2*c*; *R*^2^ = 0.015, *p* = 0.51), body mass (figure 2*d*; *R*^2^ = 0.017, *p* = 0.48) and mass-specific metabolic rate (figure 2*e*; *R*^2^ = 0.038, *p* = 0.30).

These results indicate that the spurious contribution of exosomal genes to the climate envelope remains possible. To control for potentially confounding effects, we performed a phylogenetic multiple regression analysis. The full model, best model and averaged model indicated that the number of genes in the functional category of *Exosome* (i.e. exosomal genes) were the main contributor to the climate envelope (table 1).

**Table 1.** The influences of explanatory variables on the climate envelope. The results of the full model, best model and averaged model are shown. (NOGF_Exosome_ denotes the number of genes in the functional category of *Exosome*. *N*_g_ and *N*_d_ represent the number of total genes and duplicated genes, respectively. *B*_c_ indicates mass-specific metabolic rate. s.e. is the standard error.)

	full model			best model			averaged model		
variable	estimate	s.e.	*p*-value	estimate	s.e.	*p*-value	estimate	s.e.	*p*-value
NOGF_Exosome_	0.70	0.18	6.3 × 10^–4^	0.70	0.14	5.3 × 10^–5^	0.74	0.17	7.8 × 10^–6^
*G*	–0.081	0.331	0.81				0.032	0.33	0.92
*N*_g_	0.27	0.19	0.16	0.23	0.12	0.054	0.24	0.14	0.080
*N*_d_	–3.7 × 10^–6^	9.9 × 10^–5^	0.97				4.1 × 10^–5^	9.7 × 10^–5^	0.67
body mass	–4.8 × 10^–3^	0.59	0.99				0.047	0.30	0.88
*B*_c_	–0.085	0.54	0.88				–0.055	0.27	0.84
AICc			97.5			84.7			

## 4. Discussion

The main result of this study is the association between the number of genes that code for proteins and RNAs in exosomes and habitat variability in mammals. The effect of the exosome is independent of the other genomic factors, physiological parameters and phylogenetic relationships. It is the number of exosomal genes rather than the other genomic and physiological factors that affects species habitat variability. A previous study [8] emphasized the importance of gene duplication to habitat variability, but limited to Euarchontoglires. However, the contribution of exosomes to habitat variability was concluded for all mammals. This fact also indicates a greater importance of exosomes to habitat range expansion.

We also confirmed that habitat variability is positively associated with the number of total genes although the number of total genes was a confounding variable. These results are near-consistent with the findings of previous studies [4,5]: that the redundancy of genes enhance biological robustness and that it increases species habitat variability. However, we could not conclude any relationship between genome size and habitat variability. This may be because the previous studies focused on prokaryotes whereas this study focused on mammals. On the other hand, we concluded that metabolic rate and body size hardly influence species habitat variability. This result suggests that the hypothesis that metabolic rate and body size determine habitat variability because of the wider (relative) space use of animals with smaller body sizes and faster mass-specific metabolic rates is barely accepted.

Although the role that exosomes play in biology is not yet well understood [32], the observed association may be because exosomes act as carriers for intercellular communication [33]. Exosomes are small endocytic vesicles that can enhance communication by transporting bioactive molecules (e.g. proteins and RNAs, including non-coding RNAs) between different cells. Cell-to-cell communication allows for the coordination of cell functions, thus plays an important role in the development and environmental adaptation of multicellular organisms [34]. The number of proteins and RNAs involved in exosomes is linked to the strength and promptness of cell-to-cell communication. That is, more regulatory genes involved in exosome circulation help to fine-tune communication between cells in a wider spectrum of environmental conditions. From these facts, we can hypothesize that the number of exosomal genes influences habitat variability in mammals.

The contribution of exosomal genes can also be discussed in a different context. Exosomes play an important role in diseases (cancer, in particular) by transporting bioactive molecules such as non-coding RNAs [35,36]. Specifically, microRNAs, which are a type of non-coding RNA, help to confer robustness to biological processes by maintaining transcriptional processes [37]. Such biological robustness may be linked to habitat variability. This also indicates the importance of exosomes to habitat variability because exosomes also transport microRNAs between cells.

Our study has some general limitations. For example, we only considered organisms for which genome sequences were completed. Moreover, our findings depended significantly on the quality of genome annotation. In particular, there are limitations to our phylogenetic comparative analysis. A phylogenetic comparative analysis assumes a Brownian motion-like evolution of biological traits on a phylogenetic tree with accurate branch lengths, which may result in a misleading conclusion. For example, statistical power decreases when a dataset is reduced in size following phylogenetic corrections [38]. In particular, our dataset contained only a few samples for mammals, so it may fall into that situation. Thus, the continued sequencing of genomes from a wide range of organisms will be important. In addition to this, the effects of genetic variation were not considered in this study because of the limited amount of available data. The development of high-throughput sequencing techniques may resolve these problems.

The definition of habitat variability is also controversial. In accordance with previous studies [7,8], we also considered climate envelope as a definition of habitat variability. However, it was limited to the context of climatic niche width. We may need to consider alternative definitions. We obtained similar conclusions when using habitat diversity [8] as defined by the Köppen–Geiger climate classification, which is based not only on temperature and precipitation but also on vegetation, instead of the climatic envelope. This was particularly true for the positive relationship between habitat diversity and the number of exosomal genes (*R*^2^ = 0.42, *p* = 8.7 × 10^–5^; electronic supplementary material, figure S2). This result reinforces the importance of exosomes to habitat variability.

To avoid inevitable technical problems [12,16] (e.g. combinatorial explosion and singularities in the regression model), we used the LASSO regression. However, our analysis might not be complete. We might have overlooked important gene functions because LASSO may pick only one or a few of the variables that are related to some extent to the response variable and then shrinks the rest to 0 when the explanatory variables are highly correlated [28]. To avoid this limitation, for example, we may have considered the elastic-net and relaxed LASSO. We did not use these methods because they required higher computational costs (i.e. more parameters needing to be tuned) and because the interpretation of the model can become difficult. Alternative statistical methods may be required for further investigation.

Despite these limitations, we believe in the importance of our finding (i.e. exosome hypothesis). This finding enhances our understanding of the evolution of habitat ranges in mammals. Moreover, it indicates the possibility of estimation and evaluation of not only disease but also species habitat variability by using exosome sequencing. Exosome sequencing is well used in medical sciences [39] (e.g. medical diagnosis). Similarly, we may be able to perform a diagnostic on ecosystems (e.g. biodiversity and extinction risk) through this sequencing. Sequencing analyses are now beginning to be applied in ecology (e.g. in population ecology [40] and identifying species–species interactions [41]). We suggest that the finding of this study be applied to these research fields.

## Supplementary Material

Table S1

## Supplementary Material

Table S2

## Supplementary Material

Figure S1

## Supplementary Material

Figure S2

## Data accessibility

The datasets supporting this article have been uploaded as the electronic supplementary material. The datasets are also deposited at Dryad (http://dx.doi.org/10.5061/dryad.15500) [42].
