# Supplementary material for: Exosomes in mammals with greater habitat variability contain more proteins and RNAs
Source: R Soc Open Sci. 2017 Apr 26;4(4):170162. doi: 10.1098/rsos.170162 (PMC5414279; doi:10.1098/rsos.170162)
Supplement: Figure S1 [file rsos170162supp3.docx]

**Figure S1. Phylogenetic tree of the mammals used in this study.**

The node labels correspond to species name.

The tree presented in the Newick format is as follows:

((Monodelphis_domestica:0.10446,Sarcophilus_harrisii:0.10559):0.12691,(((Rattus_norvegicus:0.08947,Mus_musculus:0.08640):0.04857,Cricetulus_barabensis:0.12545):0.09360,(Heterocephalus_glaber:0.14606,(Oryctolagus_cuniculus:0.13935,(Tupaia_belangeri:0.13140,((Callithrix_jacchus:0.04341,(((Pongo_abelii:0.01256,Nomascus_leucogenys:0.01090):0.00232,(Gorilla_gorilla:0.00644,(Pan_paniscus:0.00360,Pan_troglodytes:0.00084):0.00491):0.00364):0.00874,(Macaca_mulatta:0.01292,Rhinopithecus_roxellana:0.00991):0.01357):0.01622):0.04929,(((((Bos_mutus:0.02756,(Pantholops_hodgsonii:0.00777,(Capra_aegagrus:0.01011,Ovis_ammon:0.00730):0.00574):0.01187):0.06046,Sus_scrofa:0.07594):0.00415,Camelus_ferus:0.07446):0.01699,((Felis_silvestris:0.00886,Panthera_tigris:0.00979):0.03651,(Canis_lupus:0.03406,(Ailuropoda_melanoleuca:0.00724,Ursus_maritimus:0.00530):0.02190):0.01121):0.02913):0.00283,(((Myotis_davidii:0.02489,Myotis_brandtii:0.01461):0.09323,Equus_quagga:0.06393):0.00117,Pteropus_alecto:0.06876):0.00252):0.02157):0.00217):0.00862):0.00389):0.02292):0.07836);
