# Supplementary material for: Exosomes in mammals with greater habitat variability contain more proteins and RNAs
Source: R Soc Open Sci. 2017 Apr 26;4(4):170162. doi: 10.1098/rsos.170162 (PMC5414279; doi:10.1098/rsos.170162)
Supplement: Figure S2 [file rsos170162supp4.docx]

**Figure S2. A scatter plot of PICs in the number of genes in the functional category Exosome (NOGF_Exosome_) versus PICs in the habitat diversity.** The solid line is the regression line (*R^2^* = 0.42, *p* = 8.7 × 10^–5^).
